# Supplementary material for: Unravelling Morphological and Topological Energy Contributions of Metal Nanoparticles
Source: Nanomaterials (Basel). 2021 Dec 22;12(1):17. doi: 10.3390/nano12010017 (PMC8746323; doi:10.3390/nano12010017)
Supplement: Supplementary file 1 [file nanomaterials-12-00017-s001.zip › nanomaterials-1517093-supplementary.pdf]

nanomaterials

Supplementary Materials

Unravelling Morphological and Topological Energy Contributions of Metal Nanoparticles

Lorena Vega [1], Francesc Viñes [1,2,*] and Konstantin M. Neyman [1,2,3]

1 Departament de Ciència de Materials i Química Física, Universitat de Barcelona, c/Martí i Franquès 1-11, 08028 Barcelona, Spain; lvega@ub.edu (L.V.); konstantin.neyman@icrea.cat (K.M.N.)
2 Institut de Química Teòrica i Computacional (IQTCUB), Universitat de Barcelona, c/Martí i Franquès 1-11, 08028 Barcelona, Spain
3 Institució Catalana de Recerca i Estudis Avançats (ICREA), Pg. Lluís Companys 23, 08010 Barcelona, Spain
* Correspondence: francesc.vines@ub.edu

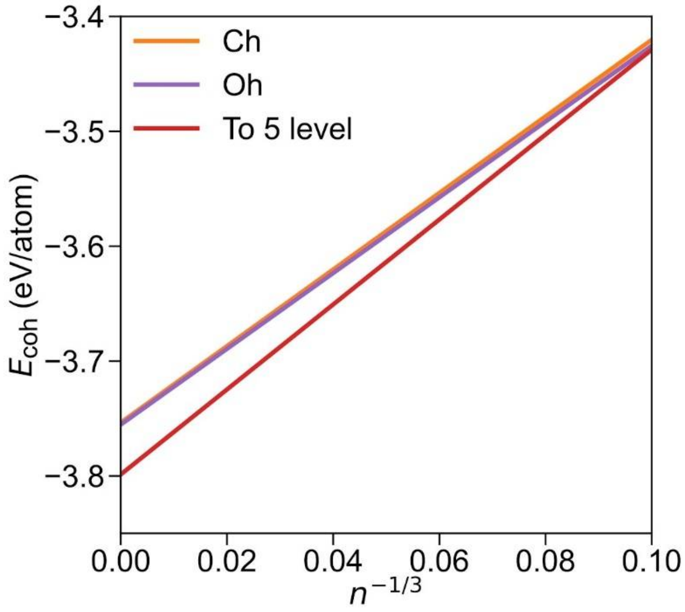

Figure S1. Comparison of most stable Cuboctahedron and Octahedron shapes and the Truncated Octahedron (To), with five levels of {001} cuts.

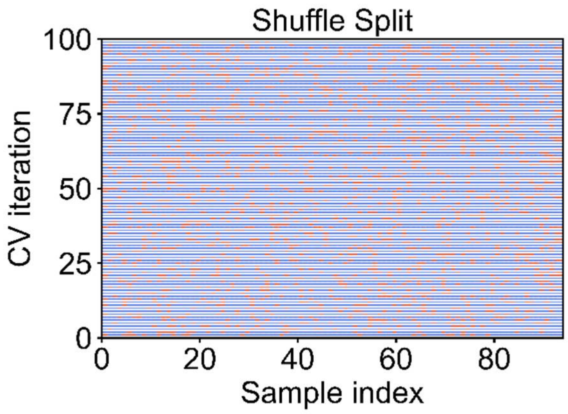

Figure S2. Visualization of each cross-validation (CV) iteration, corresponding to each of the m = 100 lines, applied using a shuffle split. Blue and orange represent training (75% of total data) and test (25% of total data) sets. Notice the random distribution of cases in between both sets.

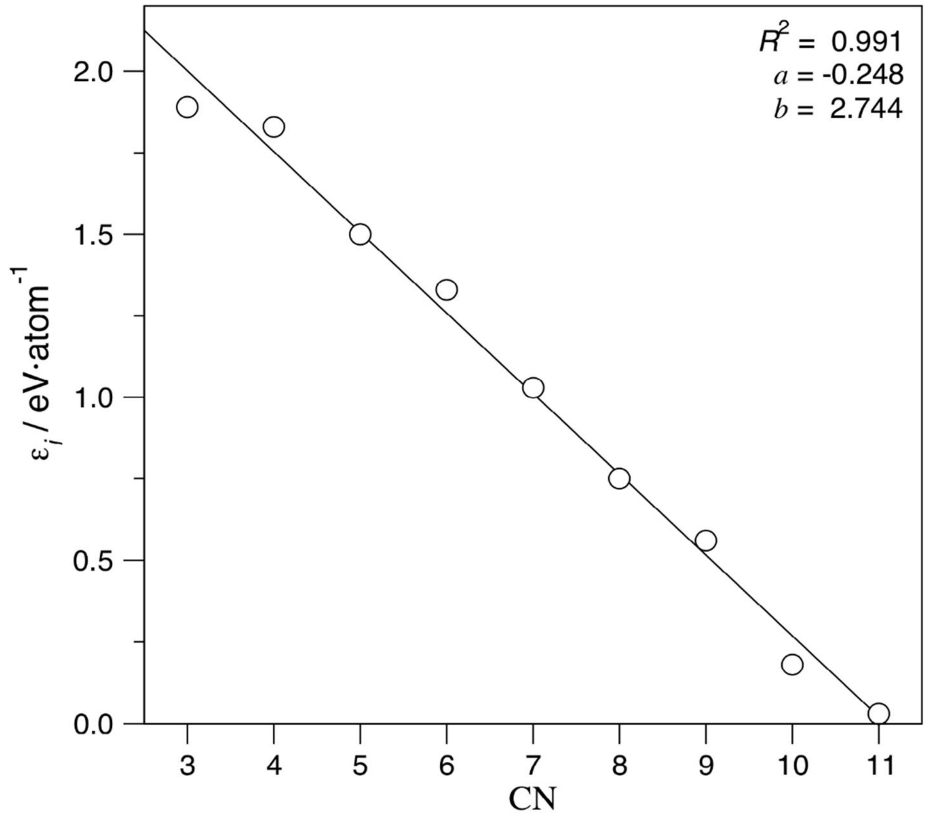

Figure S3. Linear adjustment coefficients of ε_i *vs.* CN, when considering Icosahedrons in the E_{coh} CN breakdown. Regression coefficient, R^2, slope, a, and intercept, b, are shown.

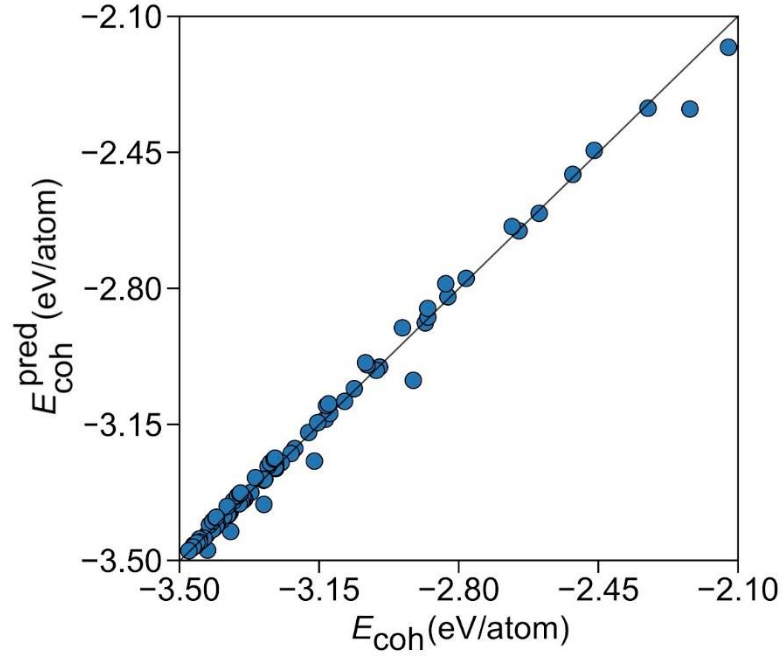

Figure S4. Comparison of calculated E_{coh} versus the predicted E_{coh}, E_{coh}^{pred}. Notice how the model overestimates the cohesion of Icosahedron shapes, with seem to follow a different linear trend.

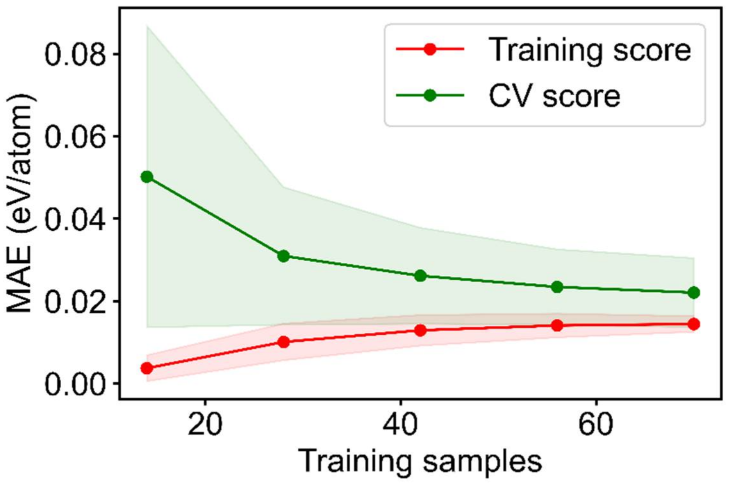

Figure S5. MAE learning curve for the training and CV scores in the E_{coh} breakdown as a matter of CN, and including Icosahedron shapes in the model. The colored areas represent the standard variation limits as a result of the 100 different fittings. .

Table S1. Summary of Pd clusters and nanoparticles according to the number of constituent Pd atoms, n, and the adopted shape, in between Spheres (S), Truncated Octahedron (To), Octahedron (Oh), Icosahedron (Ih), Cube (C), Truncated Cube (Tc), Cuboctahedron (Ch), Tetrahedron (Th), and Decahedron (Dh).

	S	To	Oh	Ih	C	Tc	Ch	Th	Dh
n	188	38	19	55	14	32	13	10	23
	290	79	44	147	63	108	55	20	54
	370	116	85	309	172	171	147	35	105
	490	140	146	561	365	340	309	56	181
	586	201	231	923	666	357	561	84	287
	682	260	344		1099	364	923	120	
	730	314	489			500	1415		
	856	338	670			610			
	976	405	891			658			
	1264	459	1156			665			
	1408	483	1469			864			
		490							
		586							
		640							
		664							
		711							
		807							
		826							
		861							
		885				994			
		976				1074			
		1072				1098			
		1126							
		1139							
		1150							
		1288							
		1289							
		1385							
		1439							
		1504							

Table S2. Average neighboring Pd-Pd distances, δ(Pd-Pd), given in Å, for each Pd_n nanoparticle, and sorted by shape. The notation in each table item is *n*(δ(Pd-Pd)).

S	To	Oh	Ih	C	Tc	Ch	Th	Dh
188(2.75)	38(2.73)	19(2.71)	55(2.76)	14(2.67)	32(2.72)	13(2.70)	10(2.66)	23(2.71)
290(2.76)	79(2.74)	44(2.73)	147(2.77)	63(2.73)	108(2.74)	55(2.73)	20(2.69)	54(2.73)
370(2.77)	116(2.75)	85(2.74)	309(2.77)	172(2.74)	171(2.75)	147(2.75)	35(2.71)	105(2.76)
490(2.77)	140(2.75)	146(2.75)	561(2.78)	365(2.76)	340(2.76)	309(2.76)	56(2.72)	181(2.77)
586(2.77)	201(2.76)	231(2.76)	923(2.78)	666(2.76)	357(2.76)	561(2.77)	83(2.73)	287(2.77)
682(2.77)	260(2.76)	344(2.76)		1099(2.77)	364(2.76)	923(2.77)	120(2.74)	
730(2.77)	314(2.76)	489(2.77)			500(2.76)	1415(2.78)		
856(2.77)	338(2.76)	670(2.77)			610(2.76)			
976(2.77)	405(2.76)	891(2.77)			658(2.76)			
1264(2.78)	459(2.77)	1156(2.77)			665(2.76)			
1408(2.78)	483(2.77)	1469(2.78)			864(2.76)			
	490(2.77)				994(2.77)			
	586(2.77)				1074(2.77)			
	640(2.77)				1098(2.77)			
	664(2.77)							
	711(2.77)							
	807(2.77)							
	826(2.77)							
	861(2.77)							
	885(2.77)							
	976(2.77)							
	1072(2.77)							
	1126(2.77)							
	1139(2.77)							
	1150(2.77)							
	1288(2.78)							
	1289(2.77)							
	1385(2.78)							
	1439(2.78)							
	1504(2.78)							

Table S3. Linear regression coefficients, R^2, as well as slopes, *a*, and intercepts, *b*, for the linear regressions of δ(Pd-Pd) *vs.* $n^{-1/3}$, according to δ(Pd-Pd) = $a \cdot n^{-1/3} + b$. Slope and intercept values are given in Å.

	a	*b*	R^2
**S**	-0.24	2.80	0.988
**To**	-0.23	2.80	0.992
**Oh**	-0.23	2.80	0.997
**Ih**	-0.14	2.79	0.997
**C**	-0.30	2.80	0.988
**Tc**	-0.23	2.79	0.994
**Ch**	-0.25	2.80	0.996
**Th**	-0.30	2.80	0.995
**Dh**	-0.31	2.82	0.988

Table S4. Atomic cohesive energy, E_{coh}, given in eV·atom^{-1}, for each Pd_n nanoparticle, and sorted by shape. The notation in each table item is $n(E_{coh})$.

S	To	Oh	Ih	C	Tc	Ch	Th	Dh
188(-3.15)	38(-2.78)	19(-2.51)	55(-2.91)	14(-2.33)	32(-2.65)	13(-2.22)	10(-2.12)	23(-2.60)
290(-3.26)	79(-3.00)	44(-2.83)	147(-3.16)	63(-2.88)	108(-3.03)	55(-2.88)	20(-2.46)	54(-2.88)
370(-3.29)	116(-3.09)	85(-3.01)	309(-3.29)	172(-3.13)	171(-3.13)	147(-3.12)	35(-2.67)	105(-3.06)
490(-3.34)	140(-3.13)	146(-3.13)	561(-3.37)	365(-3.26)	340(-3.28)	309(-3.26)	56(-2.83)	181(-3.18)
586(-3.35)	201(-3.21)	231(-3.22)	923(-3.43)	666(-3.35)	357(-3.27)	561(-3.34)	83(-2.94)	287(-3.26)
682(-3.38)	260(-3.24)	344(-3.29)		1099(-3.41)	364(-3.26)	923(-3.41)	120(-3.03)	
730(-3.39)	314(-3.29)	489(-3.34)			500(-3.31)	1415(-3.46)		
856(-3.41)	338(-3.29)	670(-3.38)			610(-3.36)			
976(-3.42)	405(-3.32)	891(-3.41)			658(-3.35)			
1264(-3.45)	459(-3.34)	1156(-3.44)			665(-3.35)			
1408(-3.46)	483(-3.34)	1469(-3.46)			864(-3.38)			
	490(-3.34)				994(-3.42)			
	586(-3.37)				1074(-3.42)			
	640(-3.38)				1098(-3.41)			
	664(-3.38)							
	711(-3.39)							
	807(-3.41)							
	826(-3.41)							
	861(-3.42)							
	885(-3.41)							
	976(-3.43)							
	1072(-3.44)							
	1126(-3.44)							
	1139(-3.44)							
	1150(-3.44)							
	1288(-3.45)							
	1289(-3.46)							
	1385(-3.47)							
	1439(-3.47)							
	1504(-3.48)							

Table S5. Linear regression coefficients, R^2, as well as slopes, *a*, and intercepts, *b*, for the linear regressions of E_{coh} *vs.* $n^{-1/3}$, according to $E_{coh} = a \cdot n^{-1/3} + b$. Slope and intercept values are given in eV·atom^{-1}.

	a	*b*	R^2
**S**	3.53	-3.78	0.994
**To**	3.26	-3.76	0.999
**Oh**	3.30	-3.76	1.000
**Ih**	3.21	-3.76	0.999
**C**	3.40	-3.74	1.000
**Tc**	3.51	-3.76	0.997
**Ch**	3.33	-3.75	1.000
**Th**	3.47	-3.74	1.000
**Dh**	3.31	-3.76	1.000

Table S6. Linear regression coefficients, *R*, as well as slopes, *a*, and intercepts, *b*, for the linear regressions of E_{coh} *vs.* $n^{-1/3}$, according to $E_{coh} = a \cdot n^{-1/3} + b$, for the Truncated Octahedron (To) subfamilies. Slope and intercept values are given in eV·atom^{-1}.

	a	*b*	R^2
**To 1 level**	3.25	-3.75	1.000
**To 2 level**	3.24	-3.76	0.999
**To 3 level**	3.32	-3.77	0.999
**To 4 level**	3.39	-3.77	0.999
**To 5 level**	3.70	-3.80	1.000

Table S7. Number of atoms for each type of CN for each studied Pd nanoparticle.

	n	3	4	5	6	7	8	9	10	11	12
**S**	188	—	—	24	24	24	—	24	24	—	68
	290	—	—	24	24	—	54	48	—	24	116
	370	—	—	—	72	48	6	8	48	24	164
	490	—	—	—	72	48	—	80	24	24	242
	586	—	—	24	72	—	72	48	48	24	298
	682	—	—	—	72	96	24	24	72	24	370
	730	—	—	—	72	72	72	48	—	72	394
	856	—	—	—	72	96	6	120	48	24	490
	976	—	—	24	48	120	54	48	72	48	562
	1264	—	—	—	120	96	24	144	24	96	760
	1408	—	—	—	120	24	120	192	—	72	880
**To**	38	—	—	—	24	—	—	8	—	—	6
	79	—	—	—	24	12	—	24	—	—	19
	116	—	—	—	24	24	6	24	—	—	38
	140	—	—	—	24	24	—	48	—	—	44
	201	—	—	—	24	36	6	56	—	—	79
	260	—	—	—	24	48	24	48	—	—	116
	314	—	—	—	24	48	6	96	—	—	140
	338	—	—	—	24	48	—	120	—	—	146
	405	—	—	—	24	60	24	96	—	—	201
	459	—	—	—	24	60	6	144	—	—	225
	483	—	—	—	24	60	—	168	—	—	231
	490	—	—	—	24	72	54	80	—	—	260
	586	—	—	—	24	72	24	152	—	—	314
	640	—	—	—	24	72	6	200	—	—	338
	664	—	—	—	24	72	—	224	—	—	344
	711	—	—	—	24	84	54	144	—	—	405
	807	—	—	—	24	84	24	216	—	—	459
	826	—	—	—	24	96	96	120	—	—	490
	861	—	—	—	24	84	6	264	—	—	483
	885	—	—	—	24	84	—	288	—	—	489
	976	—	—	—	24	96	54	216	—	—	586
	1072	—	—	—	24	96	24	288	—	—	640
	1126	—	—	—	24	96	6	336	—	—	664
	1139	—	—	—	24	108	96	200	—	—	711
	1150	—	—	—	24	96	—	360	—	—	670
	1288	—	—	—	24	120	150	168	—	—	826
	1289	—	—	—	24	108	54	296	—	—	807
	1385	—	—	—	24	108	24	368	—	—	861
	1439	—	—	—	24	108	6	416	—	—	885
	1504	—	—	—	24	120	96	288	—	—	976
**Oh**	19	—	6	—	—	12	—	—	—	—	1
	44	—	6	—	—	24	—	8	—	—	6
	85	—	6	—	—	36	—	24	—	—	19

	146	—	6	—	—	48	—	48	—	—	44
	231	—	6	—	—	60	—	80	—	—	85
	344	—	6	—	—	72	—	120	—	—	146
	489	—	6	—	—	84	—	168	—	—	231
	670	—	6	—	—	96	—	224	—	—	344
	891	—	6	—	—	108	—	288	—	—	489
	1156	—	6	—	—	120	—	360	—	—	670
	1469	—	6	—	—	132	—	440	—	—	891
**Ih**	55	—	—	—	12	—	30	—	—	—	13
	147	—	—	—	12	—	60	20	—	—	55
	309	—	—	—	12	—	90	60	—	—	147
	561	—	—	—	12	—	120	120	—	—	309
	923	—	—	—	12	—	150	200	—	—	561
**C**	14	8	—	—	—	—	6	—	—	—	—
	63	8	—	12	—	—	30	—	—	—	13
	172	8	—	24	—	—	78	—	—	—	62
	365	8	—	36	—	—	150	—	—	—	171
	666	8	—	48	—	—	246	—	—	—	364
	1099	8	—	60	—	—	366	—	—	—	665
**Tc**	32	4	—	12	—	—	12	—	—	—	4
	108	4	—	24	—	—	48	—	—	—	32
	171	—	—	36	—	—	72	—	—	—	63
	340	—	—	24	—	48	96	8	—	—	164
	357	—	—	36	—	24	126	—	—	—	171
	364	—	—	48	—	—	144	—	—	—	172
	500	4	—	48	—	—	192	—	—	—	256
	610	—	—	24	—	72	150	24	—	—	340
	658	—	—	48	—	24	222	—	—	—	364
	665	—	—	60	—	—	24	—	—	—	365
	864	4	—	60	—	—	300	—	—	—	500
	994	—	—	24	—	96	216	48	—	—	610
	1074	—	—	48	—	48	312	8	—	—	658
	1098	—	—	72	—	—	360	—	—	—	666
**Ch**	13	—	—	12	—	—	—	—	—	—	1
	55	—	—	12	—	24	6	—	—	—	13
	147	—	—	12	—	48	24	8	—	—	55
	309	—	—	12	—	72	54	24	—	—	147
	561	—	—	12	—	96	96	48	—	—	309
	923	—	—	12	—	120	150	80	—	—	561
	1415	—	—	12	—	216	120	120	—	—	923
**Th**	10	4	—	—	6	—	—	—	—	—	—
	20	4	—	—	12	—	—	4	—	—	—
	35	4	—	—	18	—	—	12	—	—	1
	56	4	—	—	24	—	—	24	—	—	4
	84	4	—	—	30	—	—	40	—	—	10
	120	4	—	—	36	—	—	60	—	—	20
**Dh**	23	—	5	—	7	—	10	—	—	—	1
	54	—	5	—	12	—	20	10	—	—	7
	105	—	5	—	17	—	30	30	—	—	23
	181	—	5	—	22	—	40	60	—	—	54
	287	—	5	—	27	—	50	100	—	—	105

Table S8. Topological factors for each studied Pd nanoparticle, including number of corners, *C*, total length of edges, *L*, total exposed surface area, *A*, and NP volume, *V*. L, A, and V are given in Å, Å², and Å³, respectively.

	n	*C*	*L*	*A*	*V*
**Oh**	19	6	62.82	94.95	67.65
	44	6	94.42	214.46	229.63
	85	6	127.46	390.83	564.93
	146	6	159.93	615.27	1115.85
	231	6	192.26	889.18	1938.63
	344	6	224.73	1214.98	3096.42
	489	6	257.44	1594.33	4654.53
	670	6	290.11	2024.71	6661.19
	891	6	323.81	2522.40	9262.51
	1156	6	356.53	3057.87	12363.35
	1469	6	390.75	3673.02	16275.80
**Ih**	55	12	166.32	266.20	371.80
	147	12	249.01	596.65	1247.62
	309	12	332.14	1061.54	2960.75
	561	12	416.15	1666.45	5823.53
	923	12	500.35	2409.00	10121.69
**C**	14	8	43.64	79.36	48.10
	63	8	89.44	333.32	414.05
	172	8	135.34	763.18	1434.54
	365	8	181.38	1370.80	3453.29
	666	8	227.92	2164.57	6852.20
	1099	8	274.69	3144.05	11995.18
**Ch**	13	12	64.84	69.08	46.48
	55	12	129.43	275.23	369.65
	147	12	195.11	625.46	1266.31
	309	12	206.81	1117.69	3025.02
	561	12	327.15	1758.49	5969.74
	923	12	393.98	2522.40	9262.51
	1415	12	461.17	3494.44	16722.91
**Th**	10	4	30.58	45.00	15.61
	20	4	47.11	106.79	57.05
	35	4	63.17	192.01	137.55
	56	4	79.37	303.07	272.77
	84	4	95.56	439.33	476.07
	120	4	111.95	602.95	765.45
**Dh**	23	7	79.55	121.79	90.49
	54	7	120.16	277.86	309.78
	105	7	160.76	497.35	736.91
	181	7	201.80	783.71	1453.53
	287	7	242.92	1135.62	2529.00

Equation S1. Resulting model correlating atomic E_{coh} as a function of the fraction of atoms, χ_i, with *i* being their CN, considering all sorts of shapes, including Icosahedrons.

$$\boldsymbol{E_{coh} = -3.73 + 1.89\chi_3 + 1.83\chi_4 + 1.50\chi_5 + 1.33\chi_6 + 1.03\chi_7 + 0.75\chi_8 + 0.56\chi_9 + 0.18\chi_{10} + 0.03\chi_{11}}$$
